# Dietary inflammatory potential and oral cancer risk: a meta-analysis of observational studies

**DOI:** 10.3389/fonc.2026.1913476

**Published:** 2026-09-11

**Authors:** Bin Li, Jie Chen, Pengkui Yu

**Affiliations:** 1 Department of Stomatology, Shengzhou People’s Hospital (Shengzhou Branch of the First Affiliated Hospital of Zhejiang University School of Medicine, the Shengzhou Hospital of Shaoxing University), Shengzhou, Zhejiang, China; 2 Department of Surgery, People’s Hospital of Jingning She Autonomous County, National Branch of the First Affiliated Hospital of Zhejiang University School of Medicine, Lishui, Zhejiang, China; 3 Department of Urology, Shengzhou People’s Hospital (Shengzhou Branch of the First Affiliated Hospital of Zhejiang University School of Medicine, the Shengzhou Hospital of Shaoxing University), Shengzhou, Zhejiang, China

**Keywords:** dietary inflammatory potential, inflammation, meta-analysis, observational studies, oral cancer

## Abstract

**Background:**

Chronic inflammation is a recognized hallmark of cancer. The association between the inflammatory potential of diet and the risk of oral cancer remains unclear. We conducted a systematic review and meta-analysis to quantify this association.

**Methods:**

A comprehensive search of PubMed, EMBASE, and Web of Science was performed to identify observational studies investigating the relationship between dietary inflammatory potential scores and oral cancer risk. Pooled odds ratios (ORs) and 95% confidence intervals (CIs) were calculated using a random-effects model.

**Results:**

Seven observational studies met the inclusion criteria. The meta-analysis revealed that individuals in the highest category of dietary inflammatory potential had a significantly higher risk of oral cancer compared to those in the lowest category (Pooled OR = 1.92, 95% CI: 1.41–2.62). The continuous analysis indicated that each 1-unit increment in the dietary inflammatory potential score was associated with a 19% increase in oral cancer risk. Sensitivity analysis using the leave-one-out approach confirmed the robustness of the pooled estimates.

**Conclusion:**

Our pooled analysis supports an association between dietary inflammatory potential and oral cancer risk. These findings suggest that dietary interventions aimed at reducing inflammation may be a viable strategy for oral cancer prevention.

## Introduction

Oral cancer represents a substantial global health challenge, characterized by high morbidity and mortality rates despite advances in therapeutic interventions. It ranks among the most common malignancies worldwide, with squamous cell carcinoma accounting for the vast majority of cases (1). While tobacco consumption and alcohol use have long been established as the predominant risk factors, a significant proportion of oral cancer cases occur in individuals without these traditional exposures (2). This observation underscores the urgent need to identify modifiable risk factors, particularly those related to lifestyle and diet, which could offer viable strategies for primary prevention.

Chronic inflammation has been increasingly recognized as a hallmark of cancer, playing a pivotal role in tumor initiation, promotion, and metastasis (3). In the context of oral carcinogenesis, persistent inflammatory states can lead to DNA damage, cellular proliferation, and angiogenesis. Diet acts as a critical environmental modulator of systemic inflammation. Certain dietary components, such as refined carbohydrates, saturated fats, and processed meats, are known to upregulate pro-inflammatory cytokines (e.g., IL-6, TNF-α), whereas others, including fiber, antioxidants, and polyunsaturated fatty acids, possess anti-inflammatory properties (4). Consequently, the overall inflammatory potential of an individual’s habitual diet may significantly influence their susceptibility to oral cancer.

To quantify this relationship, the Dietary Inflammatory Index (DII) was developed as a validated tool to assess the inflammatory potential of diet based on a comprehensive review of the literature linking specific dietary parameters to inflammatory markers (4). Unlike traditional nutrient-based approaches, the DII provides a composite score that reflects the overall inflammatory nature of the diet. Epidemiological studies have linked a pro-inflammatory diet, indicated by higher DII scores, to an increased risk of various malignancies, including colorectal, breast, and gastric cancers (5–7).

However, the specific association between dietary inflammatory potential and oral cancer risk remains less definitive and somewhat controversial. While several observational studies have suggested that a pro-inflammatory diet increases the risk of oral cancer, others have reported null or weak evidence (8–10). These discrepancies may be attributed to variations in study design, sample sizes, geographical locations, and methodological differences in dietary assessment. Therefore, we conducted this systematic review and meta-analysis to integrate existing epidemiological evidence and clarify the association between the dietary inflammatory potential and the risk of oral cancer.

## Methods

### Search strategy and study selection

A systematic literature search was conducted to identify relevant studies investigating the association between the dietary inflammatory potential and oral cancer risk. We searched the PubMed, EMBASE, and Web of Science databases for articles published from inception up to August 2, 2026. The literature search combined controlled vocabulary (where applicable) and free-text terms related to the dietary inflammatory potential and oral cancer. The main search strategy included the following terms: “Dietary Inflammatory Index,” “DII,” “dietary inflammatory score,” “inflammatory potential of diet,” “pro-inflammatory diet,” “diet-related inflammation,” “oral cancer,” “oral neoplasm,” and “mouth cancer.” The reference lists of all eligible studies and relevant reviews were also manually screened to identify additional potentially eligible studies. This systematic review and meta-analysis was conducted and reported in accordance with the PRISMA 2020 Statement (11). The study protocol was not prospectively registered.

After removing duplicates, two independent reviewers (BL and JC) screened the titles and abstracts of the retrieved records. Full-text articles were subsequently assessed for eligibility based on the inclusion and exclusion criteria. Any discrepancies were resolved through discussion or consultation with a third reviewer.

### Eligibility criteria

Studies were included in the meta-analysis if they met the following criteria: 1) Study design: observational studies, including cohort and case-control designs; 2) Exposure: dietary intake was assessed using a validated Food Frequency Questionnaire (FFQ), and the inflammatory potential of diet score was calculated and reported; 3) Outcome: the incidence of oral cancer was confirmed via pathological or histological examination; 4) Data reporting: the study reported odds ratios (ORs), relative risks (RRs), or hazard ratios (HRs) with corresponding 95% confidence intervals (CIs) for the association between inflammatory potential of diet and oral cancer. Studies were excluded if they were reviews, editorials, animal studies, or lacked enough data to estimate the effect size.

### Data extraction and quality assessment

Data were extracted from the included studies using a standardized data extraction form. The following information was collected: first author’s name, publication year, country/region, study design, sample size (number of cases and controls/cohorts), participant characteristics (age, sex), dietary assessment method, and adjusted effect estimates with 95% CIs. We prioritized the most fully adjusted models from multivariate analyses. For studies investigating broader cancer categories (e.g., head and neck cancer or upper aerodigestive tract cancer), only the site-specific effect estimates for oral cavity cancer were extracted and included in the meta-analysis, whereas data for other anatomical subsites were excluded.

The methodological quality of the included studies was assessed using the Newcastle-Ottawa Scale (NOS). The NOS evaluates studies based on three domains: selection of study groups, comparability of groups, and ascertainment of exposure (for case-control) or outcome (for cohort). Studies were categorized as high quality (NOS score ≥ 7) or moderate/low quality (NOS score < 7).

### Statistical analysis

All statistical analyses were performed using Stata software (version 12.0, StataCorp, College Station, TX, USA). The pooled ORs and corresponding 95% CIs were calculated to estimate the association between the highest versus lowest categories of dietary inflammatory potential scores and oral cancer risk. Given the anticipated clinical and methodological diversity among studies, a random-effects model (DerSimonian and Laird method) (12) was employed to pool the effect sizes.

Statistical heterogeneity across studies was assessed using the Cochran’s *Q* test and the *I²* statistic (13). An *I²* value > 50% or a *P*-value < 0.10 for the *Q* test indicated significant heterogeneity. To explore potential sources of heterogeneity, subgroup analyses were conducted based on study design (case-control vs. cohort), geographic region (Asia vs. Europe vs. Americas), sample size (< 200 vs. ≥ 200 cases), publication year (< 2020 vs. ≥ 2020), study quality (NOS score < 7 vs. ≥ 7), dietary inflammatory potential calculation method (inflammatory diet index vs. dietary inflammatory index), and number of adjusted variables (≥ 5 vs. < 5). Meta-regression analyses were carried out to explore sources of heterogeneity.

Sensitivity analysis was conducted using a leave-one-out approach to assess the influence of individual studies on the pooled results. Publication bias was evaluated visually using funnel plots and statistically using Begg’s rank correlation test (14) and Egger’s linear regression test (15). A *P*-value < 0.05 was considered statistically significant. Additionally, the trim-and-fill method (16) was used to adjust for potential publication bias if asymmetry was detected. A continuous analysis was performed to evaluate the association between a 1-unit increment in the inflammatory potential score and oral cancer risk.

## Results

### Study search and characteristics

**Figure 1:**
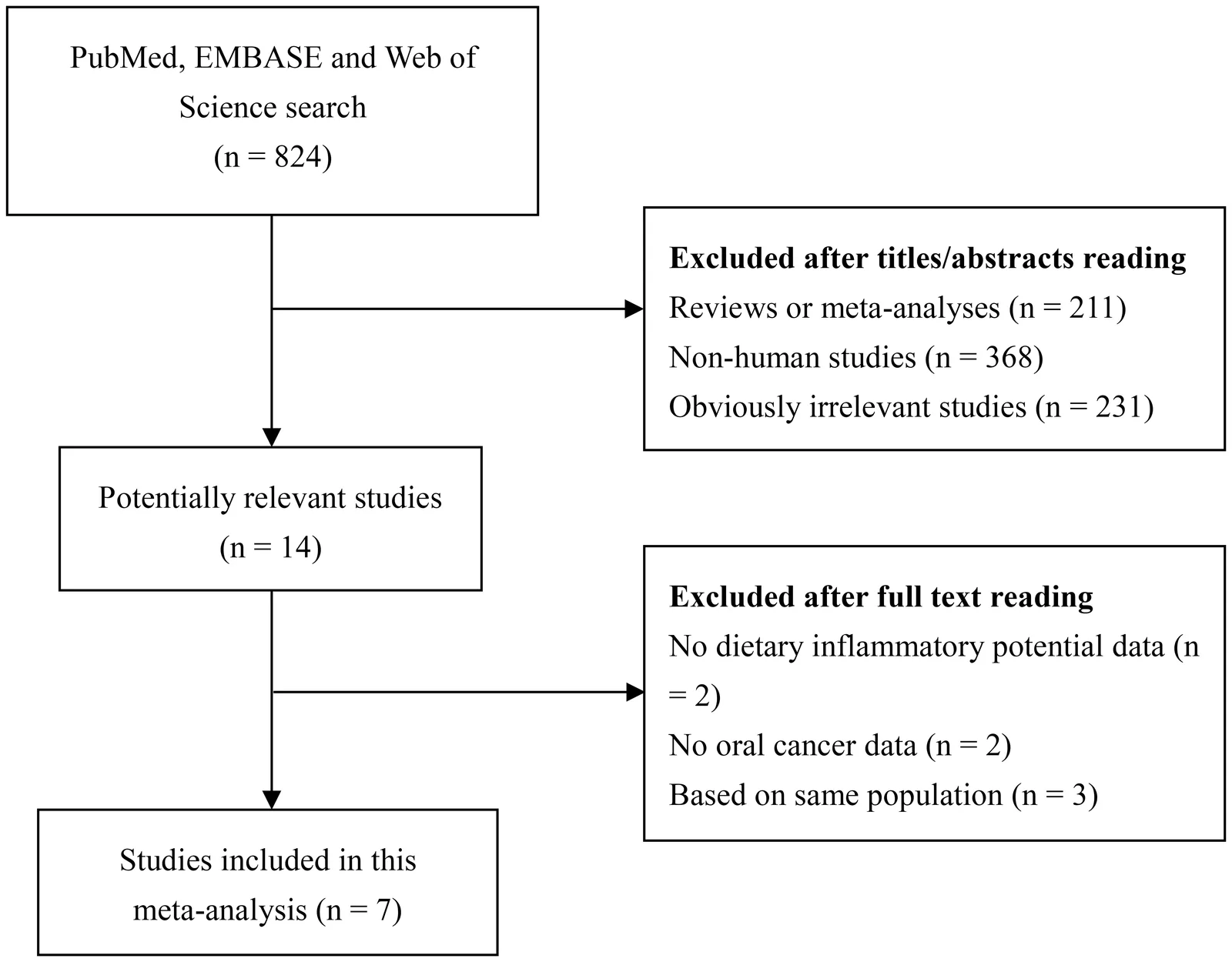
PRISMA flow diagram of the study selection process.

A systematic literature search was conducted using the PubMed, EMBASE, and Web of Science databases. The flowchart detailing the study selection process is presented in **Figure 1**. Following the screening of titles, abstracts, and full texts, a total of 7 studies (8–10, 17–20) were ultimately included in the qualitative synthesis and meta-analysis.

As summarized in **Table 1**, the analysis encompassed a total of 6 case-control studies and 1 cohort study, published between 2017 and 2024. Geographically, the studies were conducted across Asia (China, Iran, Japan), Europe (Italy, UK), and the Americas (USA, Argentina). Sample sizes varied significantly, ranging from 27 to 295 cases with control groups or cohorts spanning from 86 to over 170,000 participants. All studies utilized FFQs to assess dietary exposure and relied on either pathological or histological confirmation to diagnose oral cancer outcomes. The methodological quality of the included studies, assessed using the NOS, ranged from 6 to 8 points. Three studies were rated as high quality (NOS ≥ 7), whereas the remaining four studies were of moderate quality (NOS = 6). No study was considered to be of low methodological quality (**Supplementary Table 1**).

**Table 1:** Main characteristics of the studies included in the meta-analysis.

Study	Year	Country	Study design	Cases	Cohort/Control	Expose assessment	Outcome assessment	Adjusted variables
Narmcheshm et al.	2024	Iran	Case-control	88	1,138	FFQ	Pathologically confirmed	Energy, age, gender, province, social economic status, tobacco use, opium use, alcohol use, dental health, physical activity
Liang et al.	2024	UK	Cohort	179	170,899	FFQ	Cancer registry	Age and sex
Bao et al.	2020	China	Case-control	295	425	FFQ	Histologically confirmed	Age, gender, occupation, education level, marital status, residence, BMI, tobacco smoking, alcohol drinking, tea consumption, family history of cancer
Secchi et al.	2019	Argentina	Case-control	27	86	FFQ	Histologically confirmed	Gender, age, alcohol and tobacco consumption
Abe et al.	2018	Japan	Case-control	255	762	FFQ	Histologically diagnosed	Age, sex, smoking, ethanol consumption, flushing phenotype, teeth and occupation group
Mazul et al.	2018	USA	Case-control	165	1,396	FFQ	Cancer registry	Education, income, smoking, total lifetime alcohol intake, age, race, and sex
Shivappa et al.	2017	Italy	Case-control	208	2,492	FFQ	Histologically confirmed	Age, sex, center, year of interview, non-alcohol energy intake, education, tobacco smoking, and alcohol drinking

FFQ, food frequency questionnaire; BMI, body mass index.

### Overall analyses

**Figure 2:**
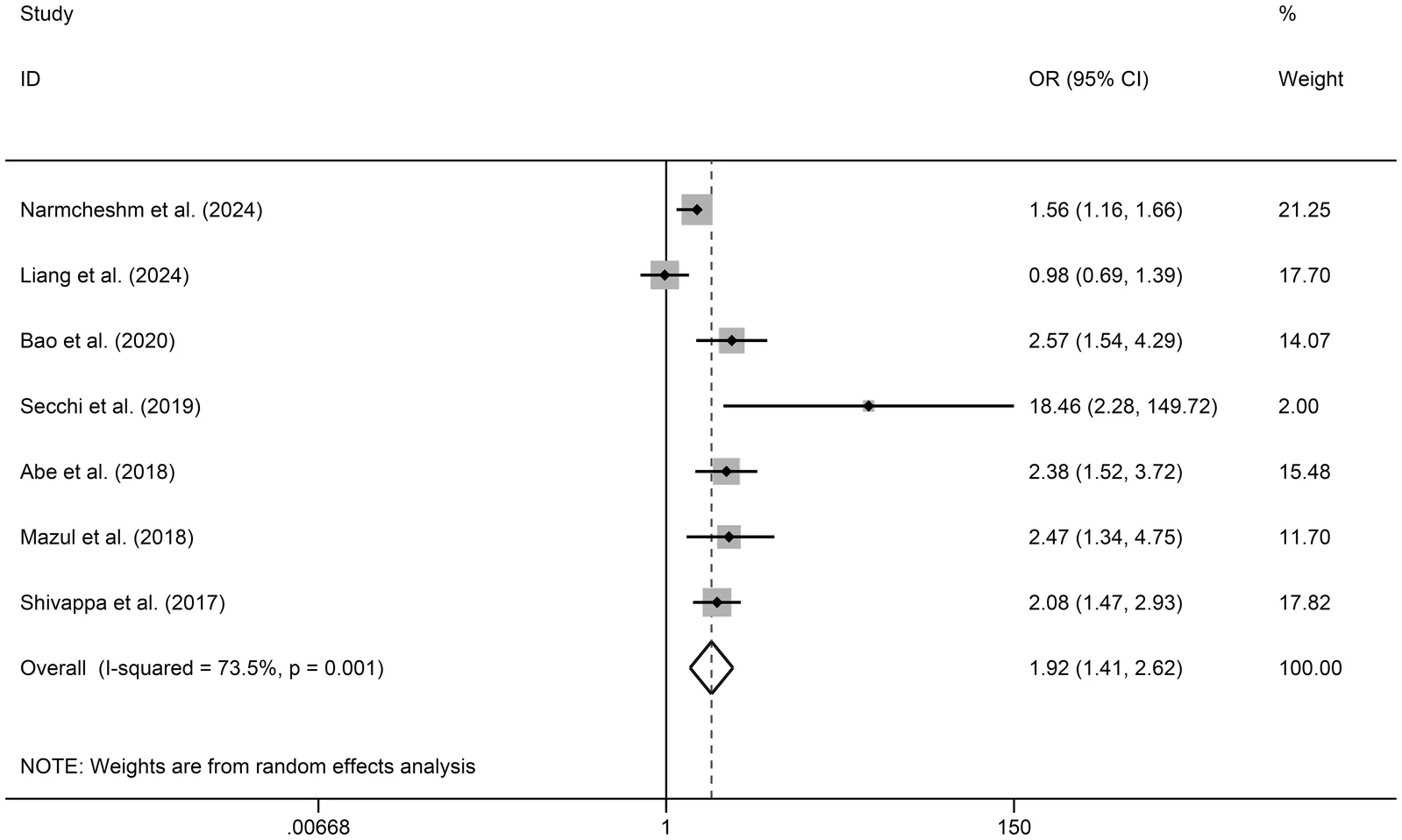
Forest plot of the association between the highest vs. lowest categories of dietary inflammatory potential and oral cancer risk. The pooled analysis used a random-effects model. The size of the square for each study is proportional to its weight in the meta-analysis. The diamond represents the overall pooled odds ratio (OR), with its width indicating the 95% confidence interval (CI).

The overall analysis, encompassing 7 studies, revealed that a higher dietary inflammatory potential score was significantly associated with an increased risk of oral cancer (**Figure 2**). The pooled OR was 1.92 (95% CI: 1.41–2.62; *P* < 0.001). This indicates that individuals with more inflammatory diets had approximately 92% higher odds of developing oral cancer compared to those with less inflammatory diets. Significant heterogeneity was observed across the included studies (*Q* = 22.67, *I²* = 73.5%; *P* = 0.001).

### Subgroup analyses

To explore the sources of heterogeneity, subgroup analyses were conducted based on study design, region, sample size, publication year, study quality, dietary inflammatory potential calculation method, and number of adjusted variables (**Table 2**). A significant positive association was observed in case-control studies (OR = 2.15), studies conducted in Asia (OR = 2.00), and in studies with a larger sample size (≥ 200 cases; OR = 2.27). The association remained significant in studies published before 2020 (OR = 2.37), using dietary inflammatory index (OR = 2.15), adjusted for at least 5 variables (OR = 2.01), and those with moderate methodological quality (NOS score < 7; OR = 2.04). Notably, the test for interaction using meta-regression analyses revealed no significant differences between any of the subgroups (all *P* for interaction > 0.05).

**Table 2:** Subgroup analyses of the association between dietary inflammatory potential and oral cancer risk.

Subgroup	Included studies	Pooled OR (95 % CI)	*P*	*P* for interaction	Heterogeneity		
					Q	I^2^ (%)	*P*
Overall	7	1.92 (1.41-2.62)	< 0.001		22.67	73.5	0.001
Study design				0.075			
Cohort	1	0.98 (0.69-1.39)	0.910		NA	NA	NA
Case-control	6	2.15 (1.62-2.85)	< 0.001		12.14	58.8	0.033
Region				0.697			
Asia	3	2.00 (1.40-2.85)	< 0.001		5.52	63.8	0.063
Europe	2	1.43 (0.68-2.99)	0.343		9.01	88.9	0.003
Americas	2	5.22 (0.78-35.09)	0.089		3.25	69.3	0.071
No. of cases				0.303			
≥ 200	3	2.27 (1.78-2.88)	< 0.001		0.52	0	0.773
< 200	4	1.69 (1.03-2.78)	0.039		13.76	78.2	0.003
Publication year				0.217			
≥ 2020	3	1.53 (0.99-2.35)	0.054		10.10	80.2	0.006
< 2020	4	2.37 (1.71-3.29)	< 0.001		4.20	28.6	0.240
NOS score				0.648			
≥ 7	3	1.78 (0.89-3.60)	0.105		12.30	83.7	0.002
< 7	4	2.04 (1.42-2.93)	< 0.001		9.23	67.5	0.026
Dietary inflammatory potential calculation method				0.075			
IDI	1	0.98 (0.69-1.39)	0.910		NA	NA	NA
DII	6	2.15 (1.62-2.85)	< 0.001		12.14	58.8	0.033
No. of adjusted variables				0.221			
≥ 5	5	2.01 (1.60-2.54)	< 0.001		7.43	46.2	0.115
< 5	2	3.52 (0.20-61.08)	0.387		7.36	86.4	0.007

OR, odds ratio; CI, confidence interval; No., number; NA, not applicable; IDI, Inflammatory diet index; DII, dietary inflammatory index.

### Publication bias and sensitivity analyses

**Figure 3:**
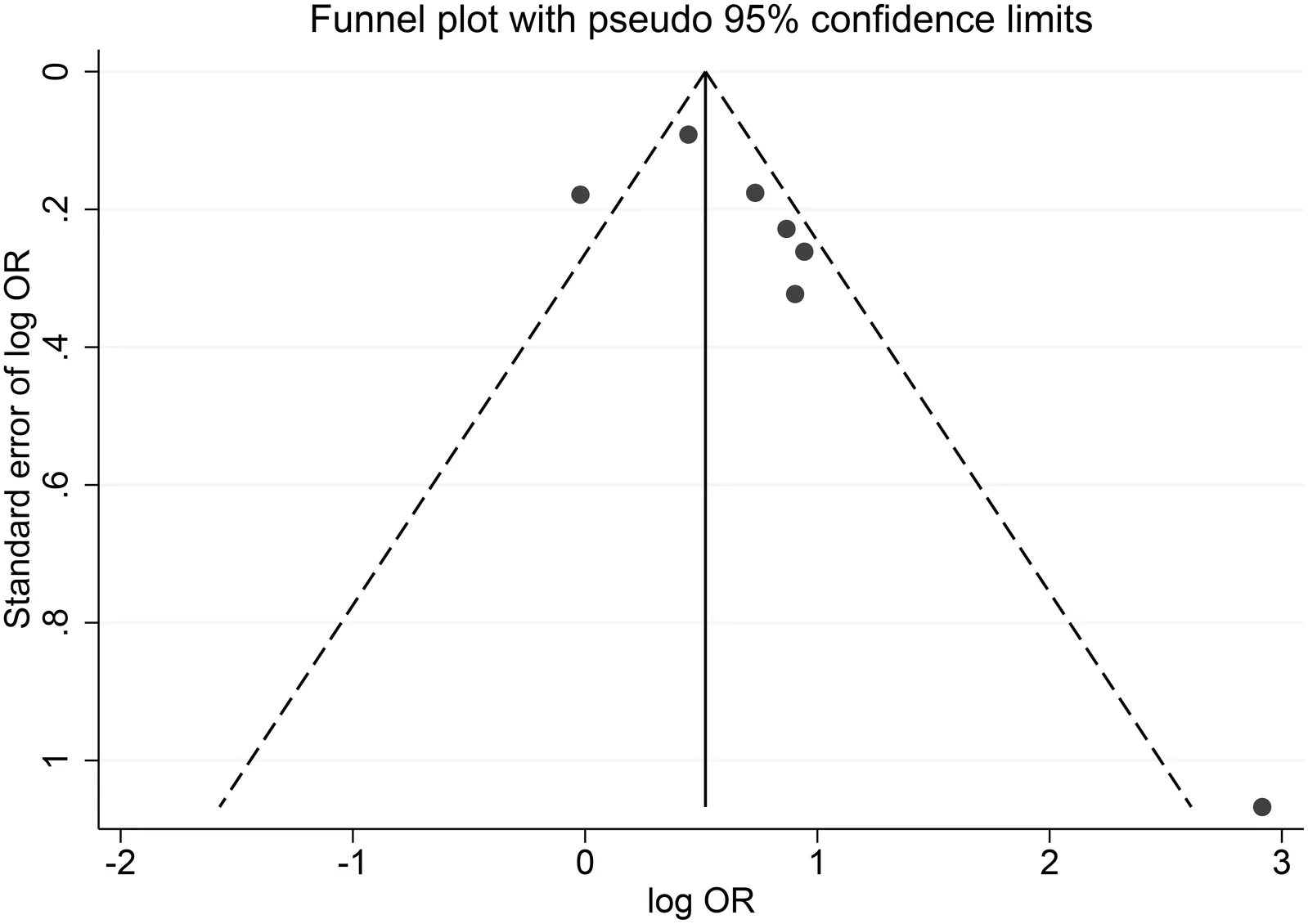
Funnel plot for the assessment of publication bias. The natural logarithm of the odds ratio (logOR) for each study is plotted against its standard error (SE).

**Figure 4:**
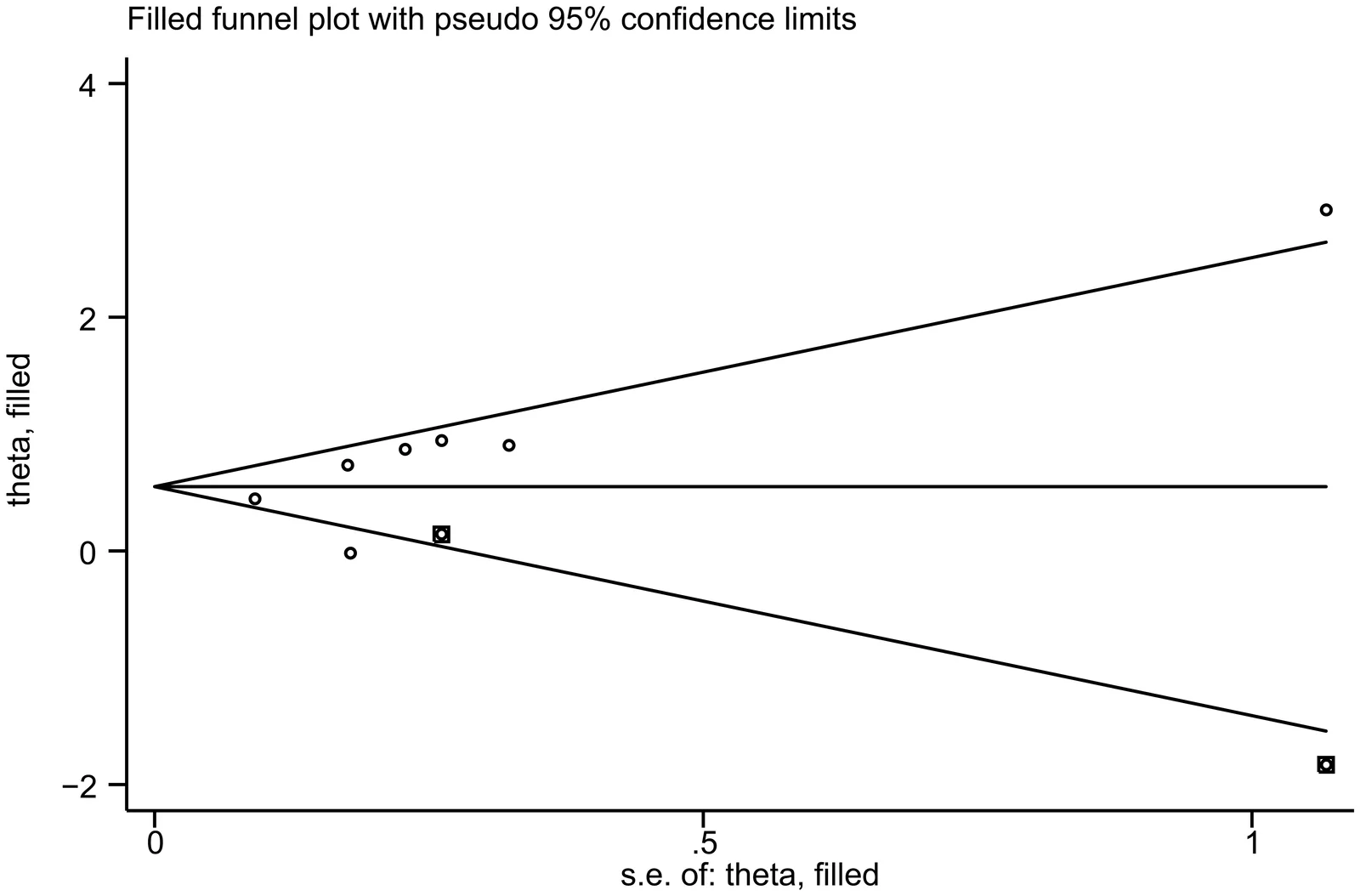
Trim-and-fill funnel plot. The squares enclosing a hollow circle represent the two imputed missing studies, and the hollow circles represent the original observed studies.

Publication bias was assessed visually using a funnel plot and statistically using Begg’s and Egger’s tests. Although neither Begg’s test (*P* = 0.133) nor Egger’s test (*P* = 0.130) indicated statistically significant publication bias, visual inspection of the funnel plot revealed a certain degree of asymmetry (**Figure 3**). Therefore, a trim-and-fill analysis was further performed to assess potential publication bias, which imputed two missing studies (**Figure 4**). The adjusted pooled effect size under the random-effects model was 1.73 (95% CI: 1.28–2.34; *P* < 0.001). The consistency of the effect direction and statistical significance before and after adjustment indicates that our findings are robust against potential publication bias.

**Figure 5:**
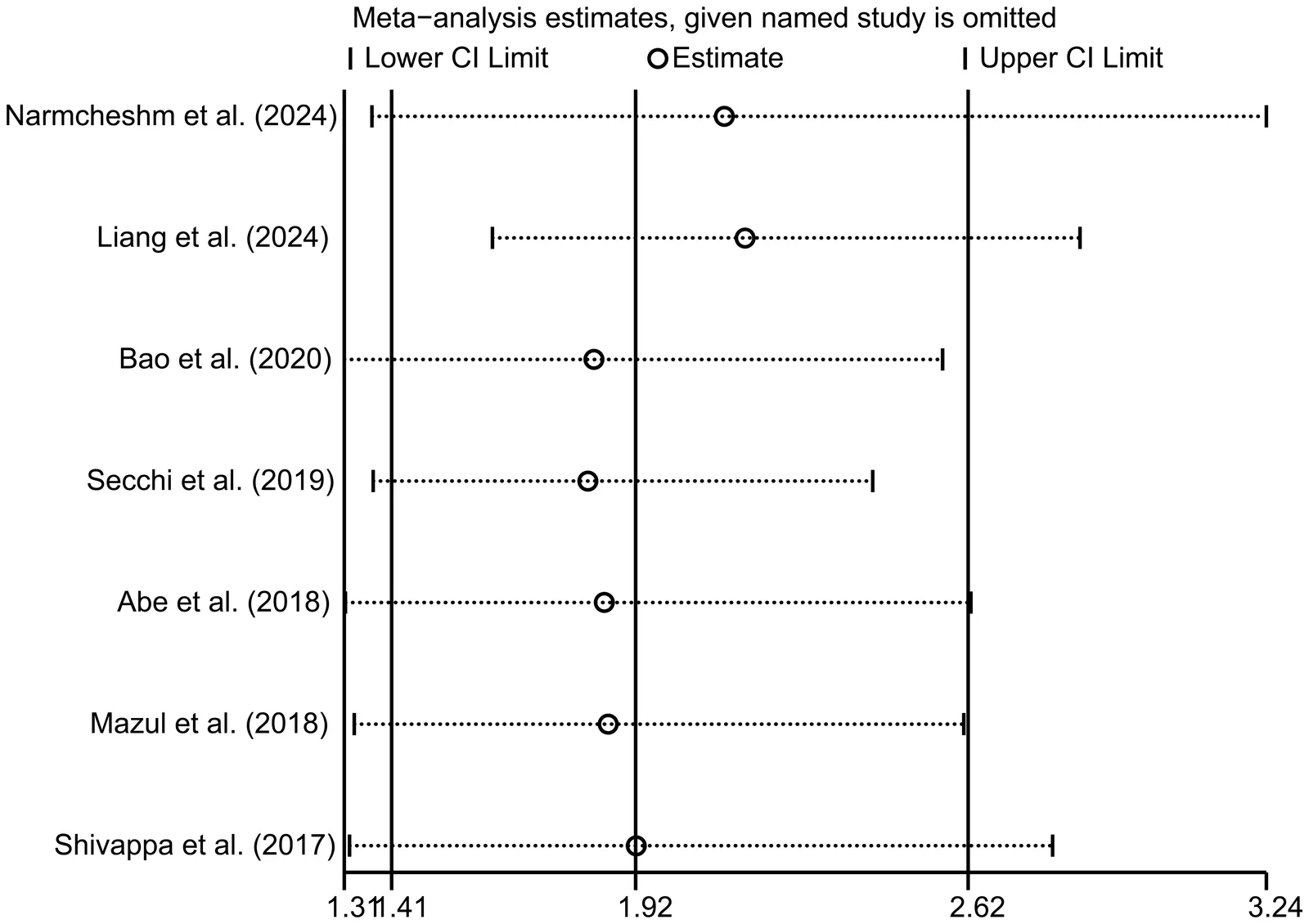
Sensitivity analysis using the leave-one-out approach. Each point represents the pooled odds ratio (OR) and its 95% confidence interval (CI) after omitting one study at a time.

A sensitivity analysis was performed using a leave-one-out approach to evaluate the stability of the pooled results (**Figure 5**). The combined effect size after sequentially omitting each individual study ranged from 1.82 (95% CI: 1.37–2.2) to 2.15 (95% CI: 1.62–2.85). This confirms that the overall results are robust and not disproportionately influenced by any single study.

### Continuous analysis

**Figure 6:**
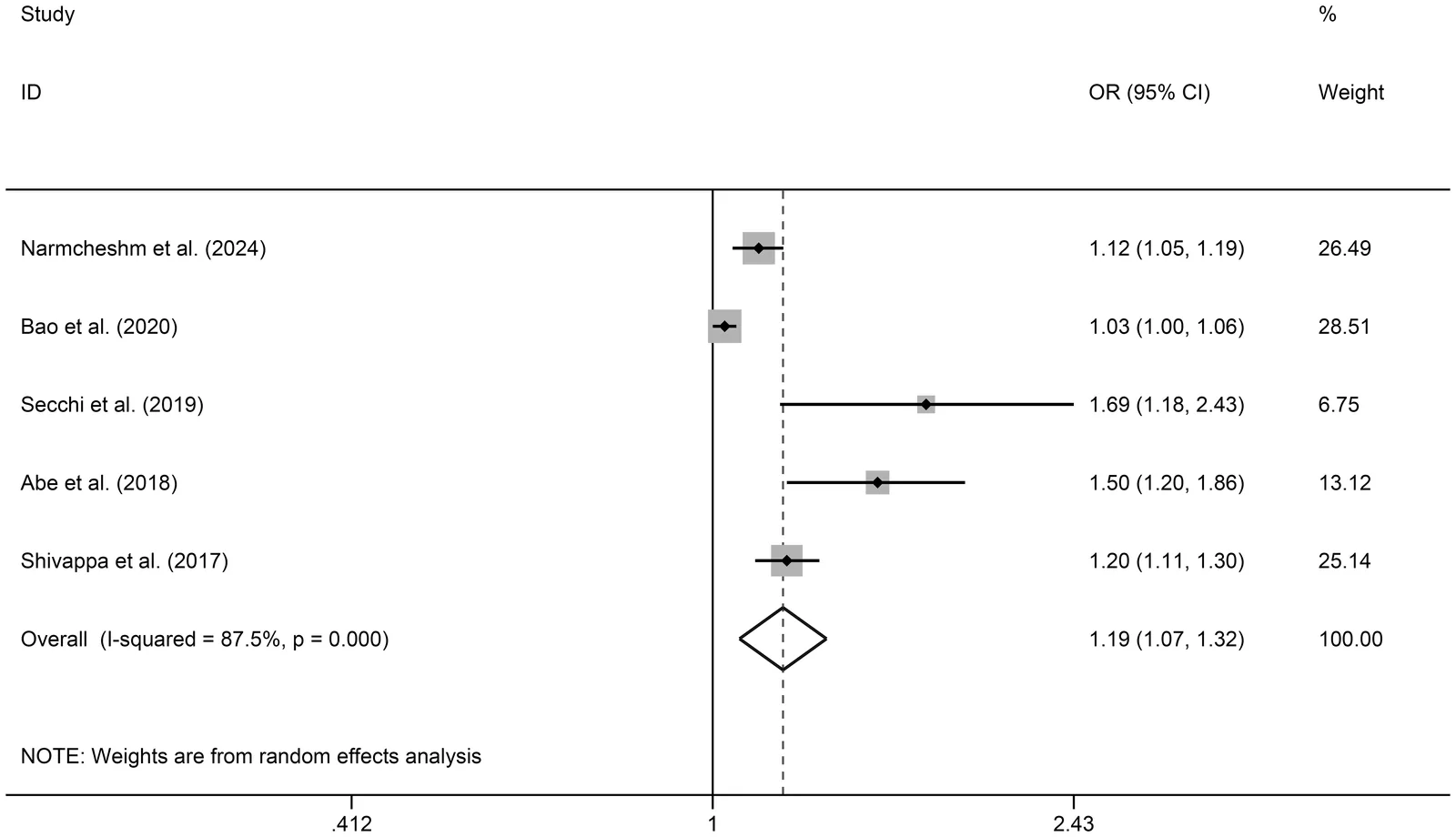
Continuous analysis of dietary inflammatory potential score and oral cancer risk. This forest plot presents the pooled odds ratio (OR) and 95% confidence interval (CI) for the risk of oral cancer associated with a 1-unit increment in the dietary inflammatory potential score. A random-effects model was used for pooling.

Five studies provided enough data to evaluate the risk associated with a 1-unit increment in the dietary inflammation score (**Figure 6**). A random-effects model was employed to pool the estimates due to the significant heterogeneity observed across the studies (I^2^ = 87.5%). The pooled analysis indicated that a 1-unit increment in the dietary inflammation score was significantly associated with an increased risk of oral cancer (Pooled OR = 1.19, 95% CI: 1.07–1.32; *P* = 0.002).

## Discussion

To the best of our knowledge, this is one of the most comprehensive meta-analyses to date evaluating the association between the dietary inflammation score and the risk of oral cancer. Our pooled analysis supports an association between dietary inflammatory potential and oral cancer risk. Specifically, individuals in the highest category of dietary inflammation scores exhibited a substantially higher risk compared to those in the lowest category. Furthermore, our continuous analysis suggests that the risk of oral cancer increases incrementally with the dietary inflammatory potential. These findings reinforce the hypothesis that diet-mediated inflammation plays a crucial role in oral carcinogenesis.

Our results are consistent with a growing body of epidemiological evidence linking pro-inflammatory diets to various malignancies. Previous meta-analyses have established strong associations between high dietary inflammation scores and cancers of the colorectum, breast, and prostate (21–23). However, the evidence specifically regarding oral cancer has been less consistent across individual studies due to sample size limitations and heterogeneity in study design. By aggregating data from multiple case-control and cohort studies, our analysis provides more precise estimates and increases statistical power, supporting an association between dietary inflammatory potential and oral cancer risk, even after adjusting for major confounders such as tobacco and alcohol use. Our findings are generally consistent with previous systematic reviews on the association between dietary inflammatory potential and oral/upper aerodigestive tract cancer (24–26). However, compared with these earlier meta-analyses, our study has several methodological advantages. Specifically, the present study incorporates more recently published evidence, includes populations from a broader range of geographic regions, and evaluates dietary inflammatory potential using the DII as well as related inflammatory dietary indices. In addition to comparing the highest versus lowest categories of dietary inflammatory potential, we performed a continuous analysis to quantitatively evaluate the change in oral cancer risk associated with increasing inflammatory diet scores. These methodological improvements provide a more reliable evaluation of the relationship between dietary inflammatory potential and oral cancer risk.

The biological plausibility of our findings is supported by the well-established link between chronic inflammation and cancer development. A pro-inflammatory diet is typically high in refined sugars, saturated fats, and processed meats, and low in fiber and antioxidants, which can lead to a state of chronic systemic inflammation. This state promotes carcinogenesis through several mechanisms: 1) Oxidative stress, chronic inflammation increases the production of reactive oxygen species (ROS), which can cause DNA damage and mutations in tumor suppressor genes (27); 2) Cytokine signaling, pro-inflammatory cytokines (e.g., IL-6, TNF-α) can activate transcription factors such as NF-κB, which regulates genes involved in cell proliferation, apoptosis inhibition, and angiogenesis (28); 3) Immune surveillance, a pro-inflammatory microenvironment may suppress effective immune surveillance, allowing malignant cells to evade detection and destruction (29).

Notably, the single cohort study from the UK Biobank (10) reported a null result, contrasting with the consistently positive findings in our pooled case-control studies. This discrepancy may be partly attributed to inherent design differences. Case-control studies are prone to selection and recall biases. Crucially, unlike the majority of our included studies that utilized the standardized, globally validated DII, the inflammatory potential of participants’ diet in this cohort was evaluated by calculating the inflammatory diet index (IDI). This methodological divergence in dietary index scoring and parameters, combined with the distinct cohort characteristics, may have reduced the statistical sensitivity required to capture the association. Although both DII and IDI aim to characterize the inflammatory potential of diet, they differ in their construction, dietary components, weighting procedures, and/or underlying derivation. Such methodological differences may partly explain the divergent effect estimates. Furthermore, the reliance on a one-time assessment of diet represents a potential source of bias. Since diet is a dynamic exposure, a single measurement may fail to reflect long-term dietary inflammatory potential, especially in cohort studies with extended follow-up durations. Such measurement error is likely to be non-differential with respect to the outcome, which tends to attenuate the risk estimates toward the null and obscure the true strength of the association.

This meta-analysis has several strengths. First, we included a large number of participants, which improved the statistical power to detect associations. Second, the use of dietary inflammatory potential score allows for a comprehensive assessment of the overall diet rather than focusing on single nutrients, which better reflects real-world dietary habits. Third, our continuous analysis further quantified the association between dietary inflammatory potential and oral cancer risk on a per-unit basis.

However, several limitations should be acknowledged. First, the primary studies included were predominantly observational (case-control and cohort designs), which precludes the establishment of a definitive causal relationship. Second, dietary intake was assessed using FFQs or 24-hour recalls, which are subject to recall bias and measurement error. Although the DII is a validated tool, misclassification of dietary exposure is possible. Third, although most included studies adjusted for major confounding factors such as smoking and alcohol consumption, the extent of covariate adjustment varied across studies. Several important risk factors for oral cancer, including human papillomavirus (HPV) infection, betel quid chewing, oral hygiene, socioeconomic status, and genetic susceptibility, were not consistently considered in all studies. Therefore, residual confounding cannot be completely excluded, and the observed association should be interpreted with appropriate caution. Fourth, only seven eligible studies were available for inclusion, limiting the overall statistical power of the meta-analysis and reducing the reliability of subgroup analyses. Moreover, because publication bias tests such as Egger’s and Begg’s have limited statistical power when fewer than ten studies are included, the absence of significant publication bias should be interpreted with caution. Fifth, although PubMed, EMBASE, and Web of Science were systematically searched and the reference lists of eligible studies were manually screened, potentially relevant studies indexed exclusively in other databases may have been missed. Finally, heterogeneity among the included studies might have influenced the pooled estimates, although we addressed this using subgroup analyses.

In conclusion, our findings supports an association between dietary inflammatory potential and oral cancer risk. This highlights the potential of dietary modification as a viable strategy for the primary prevention of oral cancer. Future large-scale prospective cohort studies are warranted to confirm these findings and to explore whether anti-inflammatory dietary interventions can effectively reduce oral cancer incidence.

## Data Availability

The raw data supporting the conclusions of this article will be made available by the authors, without undue reservation.
