# Supplementary material for: Dietary inflammatory potential and oral cancer risk: a meta-analysis of observational studies
Source: Front Oncol. 2026 Sep 11;16:1913476. doi: 10.3389/fonc.2026.1913476 (PMC13612315; doi:10.3389/fonc.2026.1913476)
Supplement: Supplementary file 1 [file Table1.docx]

**Supplementary Table 1. Methodological quality of studies included in the meta-analysis**

| **Case-control study ^1^** | **Definition**  **of cases** | **Representativeness**  **of cases** | **Selection**  **of controls** | **Definition of**  **controls** | **Comparability ^2^** | **Exposure**  **assessment** | **Same method of ascertainment for all subjects** | **Non-response**  **rate ^3^** | **Quality**  **scores** |
| --- | --- | --- | --- | --- | --- | --- | --- | --- | --- |
| Narmcheshm et al. | ☆ | ☆ | - | ☆ | ☆☆ | - | ☆ | - | 6 |
| Bao et al. | ☆ | ☆ | - | ☆ | ☆☆ | ☆ | ☆ | - | 7 |
| Secchi et al. | ☆ | ☆ | - | ☆ | ☆☆ | - | ☆ | - | 6 |
| Abe et al. | ☆ | ☆ | - | ☆ | ☆☆ | - | ☆ | - | 6 |
| Mazul et al. | ☆ | ☆ | ☆ | ☆ | ☆☆ | - | ☆ | ☆ | 8 |
| Shivappa et al. | ☆ | ☆ | - | ☆ | ☆☆ | - | ☆ | - | 6 |
| **Cohort study ^1^** | **Representativeness of the exposed**  **cohort** | **Selection of the**  **unexposed**  **cohort** | **Ascertainment**  **of exposure** | **Outcome of**  **interest not**  **present at**  **baseline** | **Comparability ^2^** | **Outcome**  **assessment** | **Follow-up long enough ^4^** | **Adequacy**  **of follow-up ^5^** | **Quality**  **scores** |
| Liang et al. | ☆ | ☆ | - | ☆ | ☆ | ☆ | ☆ | ☆ | 7 |

^1^ A study can be awarded a maximum of one star for each item except the item ‘Comparability’.

^2^ A maximum of two stars can be awarded for this item. Studies controlling for or matching by age received one star while studies additionally controlling for other important confounders received an additional star.

^3^ One star was assigned if there was no statistically significant difference in the response rate between controls and cases using a chi-square test (*P* > 0.05).

^4^ A cohort study with a follow-up time more than 10 years was assigned one star.

^5^ A cohort study with a follow-up rate more than 75% was assigned one star.
